# Saturation Dependence of Flame Thermometry Using Mid-IR Degenerate Four Wave Mixing

**DOI:** 10.1177/0003702820955186

**Published:** 2020-10-06

**Authors:** Rasmus L. Pedersen, Anna-Lena Sahlberg, Dina Hot, Zhongshan Li

**Affiliations:** Division of Combustion Physics, Lund University, Lund, Sweden

**Keywords:** Saturation, flame, thermometry, infrared degenerate four-wave mixing, IR-DFWM, water

## Abstract

It has previously been demonstrated that the ratio of the degenerate four wave mixing signal from two hot water line groups near 3231 cm^–1^ can be used for seedless flame temperature measurements. This paper presents an investigation of the impact of saturation effects on the measured signal intensity from each line group, as well as an estimation of the accuracy of the method. The saturation effects observed here would result in a large systematic error if they are not taken into account when using the degenerate four-wave mixing intensity of these water line groups to calculate the flame temperature.

## Introduction

Temperature measurements are of high importance in combustion research. Molecular line strengths are sensitive to temperature, so accurate temperature measurements are needed to accurately measure the concentration of molecular species. A variety of laser techniques have been developed for temperature measurements in combustion environments.^1^ In this work, we use the relative intensity of two water line groups to determine the temperature. This method was first proposed by Sun et al.^2^ The two water line groups in question are located in the 3230–3232 cm^–1^ range, and their relative intensity changes rapidly with temperature in the range 1000–2000 K, making this a potentially very sensitive method for flame thermometry.

The work in our laboratory has been focused on the use of infrared polarization spectroscopy (IRPS) and infrared degenerate four wave mixing (DFWM) for combustion diagnostics of molecular species which otherwise lack available absorption lines in the visible/ultraviolet spectral range. This includes many hydrocarbons and toxic pollutants such as CH_4_, C_2_H_6_, HCN, HCl, HF, etc. Quantitative concentration measurements in combustion environments using DFWM and IRPS have been demonstrated using calibration in a room-temperature gas flow with known concentrations.^3^ This method requires accurate knowledge of the temperature during the measurement. A major advantage of using water line thermometry (WALTHER) to determine the temperature is that the strength of the water line groups can also be measured using IRPS or DFWM. This drastically reduces the time and effort needed to obtain the temperature during the concentration measurement, and makes it easier to ensure that the temperature and concentration measurements are taken under the same conditions. For these reasons, WALTHER was employed for spatially resolved HCN concentration measurements.^4^

The effect of saturation was already investigated in Sun et al.^2^ However, a recent work has raised new questions. Firstly, we have suspected that the mid-infrared dye-pumped laser system, used for IRPS and DFWM measurements, jumps between different longitudinal modes in a way that can vary from scan to scan and thus make the results less reproducible. The dye laser system (Sirah, PRSC-D-18) has recently been equipped with a dynamic mode operation system (DMO), which vibrates the dye laser cavity to increase the randomness of the longitudinal mode structure. It would be good to investigate how this improves the accuracy and repeatability of the measurements. Secondly, the effects of saturation on the accuracy of the WALTHER method need to be quantified. Finally, we wished to investigate how the on-line approach to WALTHER used in Hot et al.^4^ perform compared to measuring the line group strength by excitation scans.

This paper performs a systematic investigation of the DFWM signal from the two water line groups employed for water line flame thermometry. The repeatability of the excitation scans and the stability of the measured signal intensity were investigated in several laminar CH_4_/H_2_/air flames at different temperatures. The saturation intensity for the different line groups was investigated at different flame temperatures, and the effect of the different degree of saturation on the WALTHER line ratio was thoroughly investigated. In order to calibrate the results, the temperature in the flames was measured using laser Rayleigh scattering (LRS), and this value was used as a reference for the later WALTHER measurements using both the on-line and scanning approach.

## Theory

### Water Line Thermometry with Degenerate Four Wave Mixing

**Figure 1.**
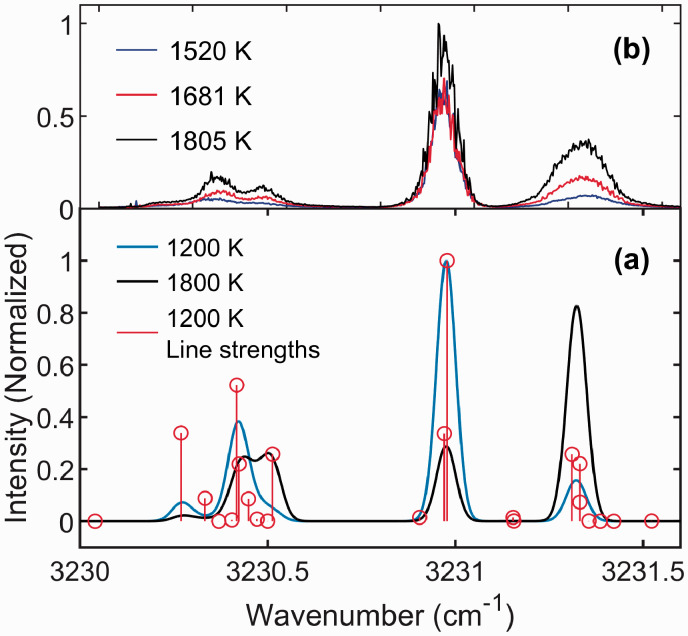
(a) Simulated DFWM water line spectra of the line groups used for thermometry. This shows the large change in ratio between line groups II and III over the primary range of the waterline thermometry technique. The red vertical lines indicate individual transition lines. The data used for the calculated line shapes was taken from HITEMP 2010.^5^ (b) Line scans measured in selected CH_4_/H_2_/air flames. As can be seen here, the change the in relative intensity between line groups II and III is very clear even for smaller changes in temperature. The pump intensity during the measurements was *I_pump_* = 0.6 TWm^–2^.

Figure 1a shows the simulated DFWM water line spectra at 1200 K and 1800 K. Each of the lines in this spectral range is in fact a line group consisting of two or more closely spaced transitions, also marked in the figure. The DFWM water line thermometry technique introduced by Sun et al.^2^ uses the ratio between water line groups II and III to determine the flame temperature. As can be seen in the figure, the relative strength of line groups II and III changes rapidly over this temperature range: line group II has highest intensity at lower temperatures, while the relative strength of line group III increases at higher temperatures. Figure 1(b) shows the measured DFWM excitation scans of these water lines recorded in laminar CH_4_/H_2_/air flames. The scans were recorded at 1520 K (Flame 8), 1681 K (Flame 5) and 1805 K (Flame 1). The flow rates of fuel and air for the different flames are shown in Table III. The measured spectra also show the increasing intensity in line group III with increasing temperature.

**Figure 2.**
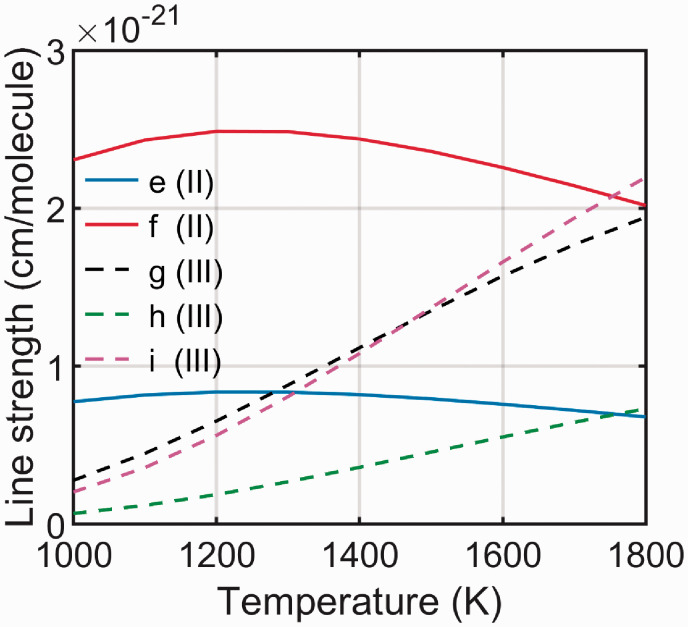
The line strength as a function of temperature for the individual lines comprising the two water line groups used for thermometry. The data used to calculate these were taken from HITEMP 2010.^5^

The individual transition lines that make up line groups II and III are listed in Table I. Note that the data used here come from HITEMP 2010,^5^ while Sun et al.^2^ used HITEMP 2000 data. The line labelled as ‘e’ in this article was not included in the HITEMP 2000 database, and therefore was not included in the analysis by Sun et al. The line strength *S_n_* of each individual line *n* scales with the temperature *T* as^6^

(1) $$S_{n}(T)=S_{n}(T_{\text{ref}})\frac{Q(T_{\text{ref}})}{Q(T)}\times \frac{e^{-E_{l,n}/k_{\text{B}}T}}{e^{-E_{l,n}/k_{\text{B}}T_{\text{ref}}}}\frac{\left[1-e^{-E_{n}/k_{\text{B}}T}\right]}{\left[1-e^{-E_{n}/k_{\text{B}}T_{\text{ref}}}\right]}$$

where *E_n_* is the energy level of the lower state of the transition, *Q* is the partition function, $k_{\text{B}}$ is the Boltzmann constant and $T_{\text{ref}}$ is the reference temperature at which the line strength is given by the HITEMP database. The temperature dependence of the line intensity of the individual transitions e–i, calculated using Eq. 1, is shown in Fig. 2.

**Table I.** Water line data for the two groups used for WALTHER, taken from HITEMP 2010.^5^

Line group	No.	Wavenumber (cm^–1^)	Line strength at 1200 K (cm/molecule)	Line strength at 1800 K (cm/molecule)	Lower state energy (cm^–1^)
II	E	3230.982970	$8.36\times 10^{-22}$	$6.79\times 10^{-22}$	1789.0428
II	F	3230.983290	$24.87\times 10^{-22}$	$20.17\times 10^{-22}$	1789.0428
III	G	3231.320581	$6.53\times 10^{-22}$	$19.43\times 10^{-22}$	5035.1265
III	H	3231.331625	$1.87\times 10^{-22}$	$7.31\times 10^{-22}$	5713.2500
III	I	3231.331625	$5.62\times 10^{-22}$	$21.95\times 10^{-22}$	5713.2500

Note: The lines are named to match the nomenclature used by Sun et al.,^2^ except for the line labelled “e”.

As can be seen, the line strength of the transitions in line group II changes very little over the interval 1000–1800 K, while the line strength of the transitions in line group III changes rapidly within this temperature range.

Saturation of the DFWM signal in the strong field limit for equal intensity pumps has been investigated by Williams et al.^7^ The line-center DFWM signal, *I_signal_*, is given by

(2) $$I_{\mathrm{signal}}=4\alpha_{0}^{2}L^{2}[\frac{I_{\mathrm{pump}}}{I_{\mathrm{sat}}}{]}^{2}[\frac{1}{1+4I_{\mathrm{pump}}/I_{\mathrm{sat}}}{]}^{3}I_{\mathrm{probe}}$$

where *I_pump_* and *I_probe_* are the pump and probe beam intensities, *L* is the length of the interaction region and *α*_0_ is the line-center attenuation coefficient calculated from the line strengths and the concentration of the absorbing species. The line-center saturation intensity, *I_sat_*, is given by

(3) $$I_{\mathrm{sat}}=\frac{ℎc{ℎ}_{0}}{2\tau_{1}\tau_{2}\mu 2}$$

where *c* is the speed of light, τ_1_ is the population dephasing rate, *τ*_2_ is the collision dephasing rate, ε_0_ is the vacuum permittivity, and *μ* is the transition dipole moment. The HITEMP line strength *S* is related to the transition dipole moment as S∝μ2. However, *S* also depends on the population of the ground state level, which changes with temperature according to the Boltzmann distribution. Assuming equal pump and probe intensity, Eq. 2 simplifies to

(4) $$I_{\mathrm{signal}}=[\frac{4\alpha_{0}^{2}L^{2}}{I_{\mathrm{sat}}^{2}}]\frac{I_{\mathrm{pump}}^{3}}{(1+4I_{\mathrm{pump}}/I_{\mathrm{sat}})3}$$

As can be seen, the line-center DFWM signal changes significantly depending on the degree of saturation. For $I_{\mathrm{pump}}\approx I_{\mathrm{sat}}$, the signal intensity is approximately linearly dependent on *I_pump_*. The reduced intensity dependence, together with the reduced sensitivity to collisional quenching effects,^8,9^ is why it is generally preferred to work at or above the saturation limit. The complication in this case is that, due to the difference in transition dipole moment, the individual transitions in line groups II and III will saturate at different pump intensities. The pump intensity must therefore be carefully considered when using the DFWM signal intensity from these line groups for flame thermometry.

The mathematical model described by Williams et al.^7^ assumes a weak probe field and saturating pump beams, while probe and pump beams of equal intensity are used here. In addition, interference effects from the closely spaced transition lines within group II and III are not included in the model. The mathematical model is, therefore, only used to provide a rough estimate of signal behaviour. Saturating probe fields in DFWM have been treated by a non-perturbative analytical model^10^ and by direct numerical simulation (DNS).^11^ Closely spaced transitions in DFWM have been treated analytically^12^ and by DNS.^13^ However, the application of these simulation methods is outside the scope of this work.

### Laser Rayleigh Scattering Thermometry

Laser Rayleigh scattering (LRS) measures the elastic scattering of light from molecules. In LRS, a laser beam is directed through the measurement medium, and a camera is aligned to measure the Rayleigh scattered light from the molecules. The intensity of the scattered light can be used for accurate temperature measurements. LRS has been applied for flame thermometry in a wide variety of alignments and environments (see e.g., literature^11–14^). The intensity *I_R_* of the Rayleigh scattered light is given by^14^

(5) $$I_{R}={\mathrm{CI}}_{0}N\sigma_{R}$$

where *C* is a calibration constant, *I*_0_ is the laser intensity, *N* is the number density and *σ_R_* is the Rayleigh cross section. The cross-section varies depending on the angle of observation, where the strongest scattering is located in a direction at a 90° angle to the laser polarization. For this case, the Rayleigh cross section $\sigma_{R,i}$ for molecule *i* is defined as^14^

(6) $$\sigma_{R,i}=\frac{4\pi 2(n_{i}-1)2}{N^{2}\lambda^{2}}$$

where *n_i_* is the refractive index for molecule *i* and *λ* is the wavelength of the laser radiation. For completely accurate cross sections, the depolarization ratio of different molecules also needs to be accounted for. However, at visible laser wavelengths, the effects of the depolarization ratio are usually negligible.^15,16^ LRS flame thermometry is achieved by comparing the scattering intensity in the flame with the scattering intensity of a known medium (usually air) measured with the same setup. The temperature is calculated from the Rayleigh scattering intensity *I_a_* and *I_f_* in the air and flame, respectively, as^14^

(7) $$T_{f}=T_{a}\frac{I_{a}}{I_{f}}\frac{\sigma_{R,f}}{\sigma_{R,a}}$$

The Rayleigh cross section for a mixture becomes a weighted sum of the cross section of each molecule with the molar concentrations. Table II shows the refractive index for the major species present in the flame, together with the calculated Rayleigh cross sections at 457 nm.

**Table II.** Refractive index (*n*) and Rayleigh cross section (*σ_R_*) at 457 nm for different gases.

Species	(*n*–1)$\cdot 10^{-3}$	$\sigma R\cdot 10^{-27}$ (cm^–2^)
N_2_	0.301^a^	1.136
O_2_	0.256^b^	0.8192
H_2_O	0.278^c^	0.9655
CO_2_	0.453^d^	2.5769

a From Peck and Khanna^18^

b From Zhang et al.^19^

c Estimated from Zetterberg et al.^17^ and Gardiner et al.^20^

d From Old et al.^21^

## Experimental Arrangement

### Degenerate Four Wave Mixing setup

The DFWM pump and probe beams were generated by a pulsed IR dye laser system, which has been described previously.^22^ The mid-infrared laser light is produced by difference-frequency mixing in a LiNbO_3_ crystal, between a narrow-band dye-laser beam at 792 nm (Sirah, PRSC-D-18, with dye LDS 798) and a single-mode 1064 nm laser beam from a Nd:YAG laser (Spectra Physics, PRO 290-10). The laser provides pulse energies up to 6.3 mJ in the 3230–3232 cm^–1^ spectral range used here, with a linewidth of less than 0.025 cm^–1^, at a repetition rate of 10 Hz. In addition to what is covered in the previous descriptions,^22^ the system has been equipped with a dynamic modulation operation (DMO) mode. This randomizes the longitudinal mode distribution of the dye laser output by vibrating the cavity mirrors at 11 Hz, in order to average out effects of mode jumping. This reduces the variability between separate scans. In practice, we did not see much difference with the DMO on/off, which could be because the mode jumping is still within the linewidth of the absorption line. The shot-to-shot variation for the online measurements on line group III is much less compared to the narrower line group II, which supports this assumption.

**Figure 3.**
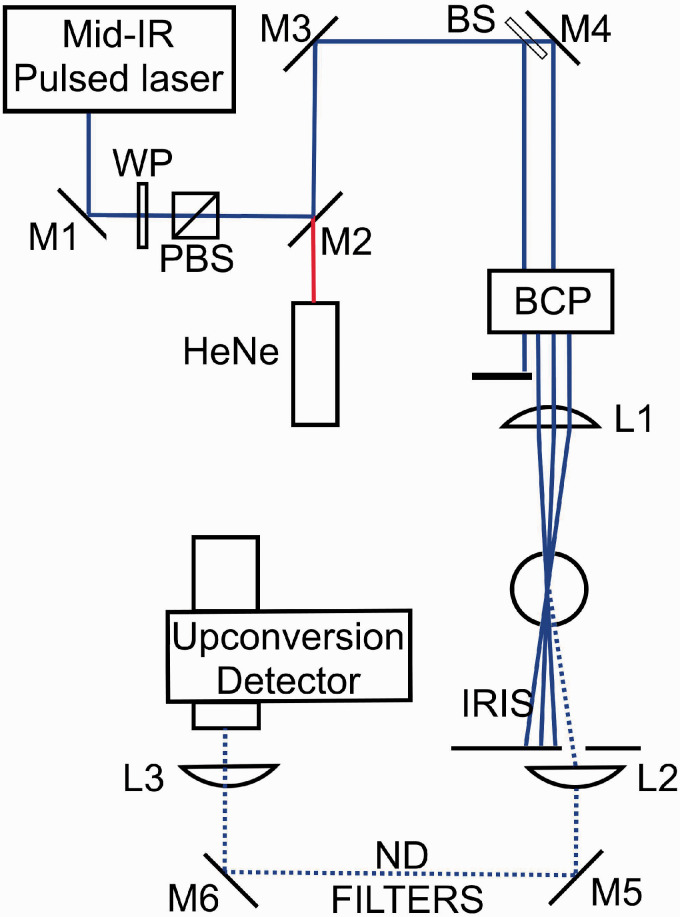
M1-7: mirrors. M2: dichroic mirror. BS: Beam splitter. PBS: polarizing beam splitter. BCP: BOXCARS plate. L1-3: lenses.

A diagram of the setup used for DFWM is shown in Fig. 3. The IR-laser is first passed through a half waveplate and a polarizing beamsplitter. The angle of the half waveplate can be adjusted to control the energy of the laser pulses transmitted through the polarizing beamsplitter. The IR laser beam is overlapped with a 632 nm visible HeNe laser beam for the ease of alignment. The IR beam is split in the horizontal plane, to two parallel beams using a beamsplitter (BS). These two beams are then split in the vertical plane into four parallel beams using a BOXCARS plate.^23^ The four beams are spaced at the four corners in a square. One of these beams is chosen to be the alignment beam. This beam traces the path the generated signal beam will follow, which enables the alignment of the signal beam path to the detector. During the experiments, the alignment beam is blocked using a beam block. The remaining beams are focused to a common crossing point using the lens L1, 2 inches in diameter with a 500 mm focal length which results in a beamwaist with a 0.22 mm radius. The size of the measurement volume is estimated to be 0.22 × 0.22 × 11 mm^3^, and the measurement point was aligned at 8 mm height above the burner (HAB). The pump and probe beams are then blocked using an iris, while the signal is let through and collimated using lens L2, which has a focal length of 500 mm. The signal is directed to the upconversion detector, where the signal intensity is reduced using ND filters if necessary. Lens L3 is used to focus the signal beam into the periodically poled lithium niobate (PPLN) crystal of the upconversion detector, to match the beam width of the 1064 nm pump in the detector. To achieve the optimal focal length, L3 consists of two lenses with focal lengths of 1000 mm and 200 mm for an effective focal length of 167 mm. The upconversion detector uses an intracavity system to do sum frequency generation between the IR signal and a 1064 nm pump, for increased sensitivity. The system and its advantages have been described in detail elsewhere.^23–25^

The signal from the water lines was recorded in two ways using this setup, either by a scanning measurement or an on-line peak measurement. The first was achieved by scanning the laser wavelength across the range 3230–3231.5 cm^–1^ at a scanning speed of 0.025 cm^–1^s^–1^ and recording the DFWM signal during the scan. Each measurement point shown in the following figures is the average of the values obtained from five scans. The standard deviation of the values from each set of five scans was used to calculate the uncertainty. The on-line method consisted of tuning the laser to the peak of a water line group and recording the signal for 30 s. The maximum value recorded during this time was used as the DFWM intensity of each line group. The maximum value was used because this provided a more consistent result compared to using the average value. One cause for this could be that the intensity data obtained for each 30 s on-line measurement does not follow a normal distribution. One on-line measurement of each peak was used to obtain the ratio, and six ratio measurements were recorded to evaluate the repeatability and precision. Scanning is generally easier to work with, and is more accurate, due to the difficulty in tuning the laser wavelength precisely to the peak wavelength. The advantage of using the on-line approach is that it allows temporal resolution for processes that change on time-scales shorter than the duration of a scan.

### Laser Rayleigh Scattering Setup

The temperature in flames 1–8 was measured using a relatively simple LRS setup. A collimated 457 nm CW laser beam, with a diameter of 2 mm and power 170 mW, was sent through the flame at 8 mm HAB. The Rayleigh scattered light (the LRS signal) was detected by an EMCCD camera (Andor Luca R DL-604M-OEM) equipped with a Nikon (50 mm, *f*/2.8) camera lens, placed at 90° to the laser beam path and to the laser polarization. The LRS signal along the laser beam path was recorded as the average of 10 images, and a reference LRS signal was recorded in a dry air flow. In addition to this, a background image was also recorded in order to remove background noise. The exposure time of the camera was set to 0.1 s, and the EM gain to 10.

### Flame

The measurements were performed in laminar, flat CH_4_/H_2_/air flames stabilized on a Perkin-Elmer burner with a plug diameter of 25 mm. Table III shows the flame composition and equivalence ratio (Φ) of the eight different flames studied here. A bluff-body stabilizer was placed 20 mm above the burner surface. The relative flows of the fuel and air were controlled by Bronkhorst mass flow controllers. A 5 L/min nitrogen co-flow was used to shield the flames.

A program called CEA (chemical equilibrium with applications)^26^ has been used to simulate the flame composition for laminar CH_4_/H_2_/air flames. Table IV shows the simulated major species concentrations in the product zone of flames 1–8, together with the flame temperatures at 8 mm HAB measured by LRS. Since the LRS thermometry requires knowledge of the flame composition, the gas composition was first simulated at an approximate temperature, and the resulting LRS temperature was used as a base for a new simulation. This was repeated until the LRS temperature was the same as the simulation temperature, within a margin of ±20 K, which took two to three iterations depending on the initial simulation temperature.

**Table III.** Fuel/air flows in flames 1–8.

	CH_4_ flow	H_2_ flow	O_2_ flow	
Flame	[l/min]	[l/min]	[l/min]	Φ
1	0.497	0	4.500	1.05
2	0.475	0	4.530	1
3	0.432	0	4.575	0.9
4	0.381	0.164	4.455	0.9
5	0.342	0.146	4.515	0.8
6	0.264	0.264	4.470	0.7
7	0.176	0.410	4.410	0.6
8	0.136	0.544	4.320	0.6

Note: The total flow rate for all flames was 5 l/min.

**Table IV.** Simulated major species mole fractions (*x_i_*) in flames 1–8, together with the flame temperature at 8 mm HAB measured using LRS.

Flame	$x_{N2}$	$x_{H2O}$	$x_{\mathrm{CO}2}$	$x_{O2}$	*T_R_*/K
1	0.7198	0.1941	0.0847	0.0014	1805
2	0.7197	0.1878	0.0878	0.0047	1781
3	0.7233	0.1725	0.0855	0.0187	1775
4	0.7203	0.1804	0.0807	0.0186	1721
5	0.7260	0.1628	0.0739	0.0373	1681
6	0.7296	0.1519	0.0612	0.0573	1638
7	0.7320	0.1443	0.0460	0.0777	1542
8	0.7275	0.1559	0.0394	0.0771	1520

## Results and Discussion

### LRS Measurements

**Figure 5.**
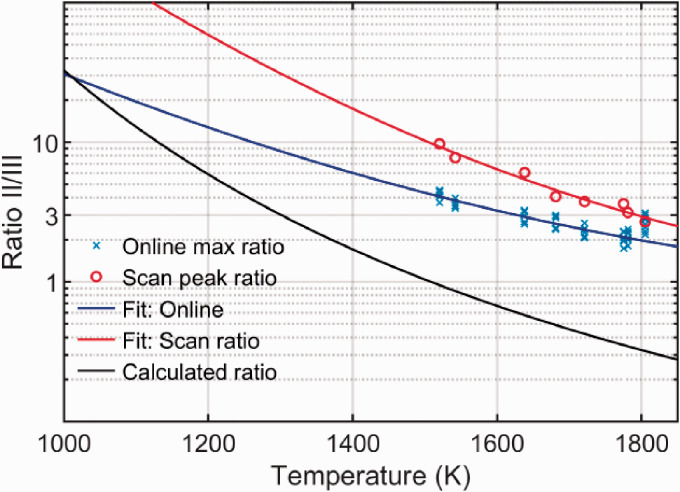
Water line ratio measurements as a function of flame temperature. The pump intensity used was *I_pump_* = 0.6 TWm^–2^.

The flame temperature measured with LRS at 8 mm HAB is shown in Table IV, and these values are used as reference temperature for the DFWM line ratios presented in Fig. 5. The uncertainty in the LRS temperature is estimated to be ±7% of the calculated product zone flame temperature. This is calculated by considering the intensity fluctuation in the Rayleigh signal intensity, as well as the uncertainty in the gas composition and the calculated Rayleigh scattering cross sections. Better precision could be achieved by a more optimized LRS setup.^27–30^

### DFWM Saturation Measurements

**Figure 4.**
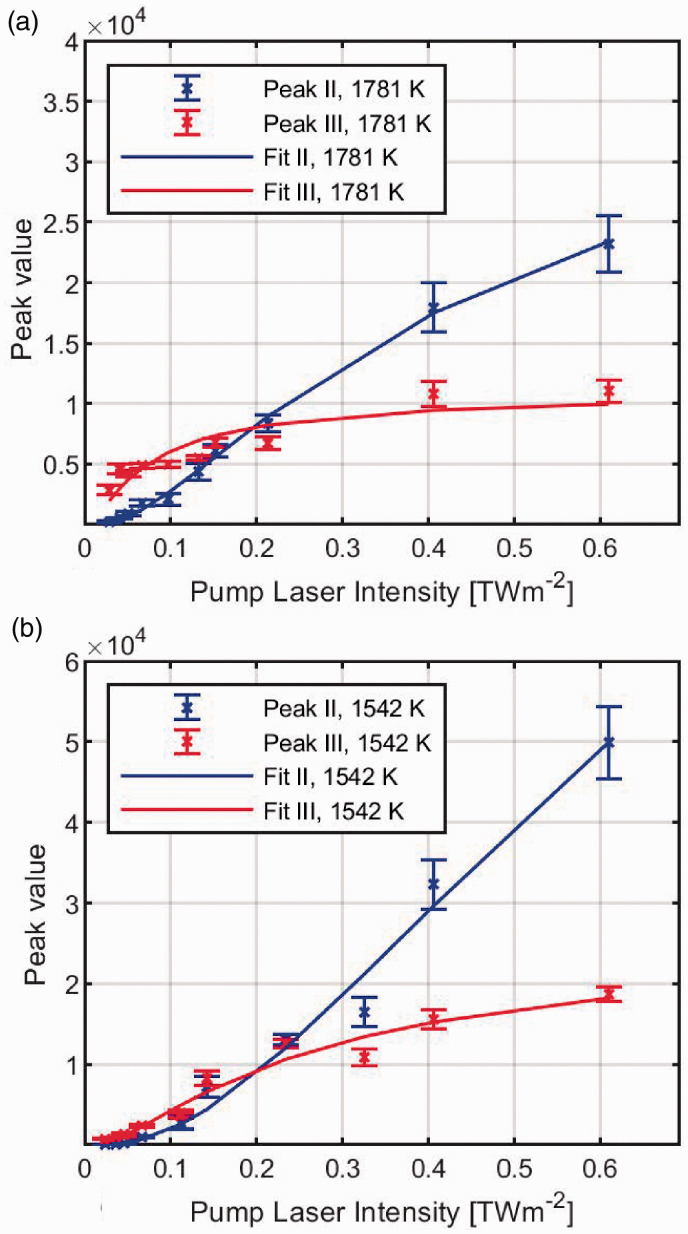
Saturation curves for the DFWM signal from line groups II and III, recorded in (a) Flame 2 (1781 K) and (b) Flame 7 (1542 K). The saturation intensity found from these curves are: $I_{\mathrm{sat},\mathrm{II}}=0.61$ TWm^–2^ and $I_{\mathrm{sat},\mathrm{III}}=88$ GWm^–2^ for Flame 2, and $I_{\mathrm{sat},\mathrm{II}}=1.48$ TWm^–2^ and $I_{\mathrm{sat},\mathrm{III}}=0.334$ TWm^–2^ for Flame 7.

To investigate the saturation behaviour of line groups II and III, DFWM excitation scans over the lines were recorded for a range of different pulse energies, in Flame 2 (1781 K) and Flame 7 (1542 K), respectively. The average peak value from five excitation scans for each line group is shown in Fig. 4 as a function of the pump beam intensity in the measurement point. Each measurement series is shown with a curve fitted to Eq. 4. From the measured peak values and matching fitting curves, it seems that group III is saturated well below the maximum pulse energy available from the current IR laser system, in contrast to group II. This is probably because group III has a higher transition dipole moment than II. Comparing the scans in Flames 2 and 7, the trend seems to indicate that the saturation intensity will be higher at lower temperatures. The difference in saturation intensity in the two flames is probably caused by the difference in collisional quenching in the flames at different temperatures, which affects the collision dephasing rate *τ*_2_. Group II approaches the saturation limit at 1781 K (Flame 2), but for 1542 K (Flame 7) this does not seem to be the case. However, for laser intensities *I* > 0.2 TWm^–2^, the intensity dependence has gone from cubic, as it is for low energies, to linear, which reduces the impact of intensity variations from pulse to pulse.

From Fig. 4, it is obvious that the intensity ratio between line groups II and III is very dependent on *I_pump_*. The peak ratios might converge to a set ratio when both groups II and III are well into the saturated regime ($I_{\mathrm{pump}}\geq 2I_{\mathrm{sat}}$), but with the saturation intensities predicted from the fits in Fig. 4, this would be a challenge to reach in practice with the current setup. In order to achieve accurate flame temperatures, it would therefore be necessary to perform a calibration measurement at known temperatures for the actual pump intensity. Such calibration measurements are shown in Fig. 5 for ratios measured by the scanning and by the on-line approach, together with a line showing the ratio calculated from HITEMP data without considering saturation. The temperature values for each flame were provided by the LRS measurements. Each measurement series was fitted with a second-order polynomial, to allow easy comparison with the trend of the calculated ratio. Within the investigated temperature interval, the ratios obtained by scanning follow the same trend as the calculated ratios, but with a constant corrective multiplicative factor caused by the saturation effects, which translates to a constant offset in the log-scale plot. Further studies are necessary to see if this multiplication factor remains constant over a wider temperature range. The on-line measurements do not follow the same trend as the scanning ratio, although why that is the case is not clear. A possible explanation is that the laser wavelength did not match the peak position perfectly; however, great care was taken to tune the laser to the position of the peak. In addition, the on-line ratio at 1805 K was consistently measured to a higher value, than what would be expected from the trend of the other on-line measurements. This outlier was repeated on two separate days of measurement, which indicates a systematic error in the way these measurements were recorded.

To compare WALTHER with other thermometry methods, it is necessary to quantify the uncertainty of the temperature measurement. This was done by calculating the temperature from the measured ratios, using the fitted calibration curves in Fig. 5, and comparing this with the temperature measured using Rayleigh scattering. The on-line outliers at 1805 K were not included in this calculation. The maximum difference between the LRS and WALTHER temperatures was 34 K and 83 K for the scanning and on-line measurements, respectively, and the average difference was 17 K and 30 K.

## Conclusion

The measurements presented show that saturation effects have major impact on the value of water line ratio. However, within the investigated temperature range and for a constant pump and probe intensity, the effect is a constant corrective multiplicative factor, at least when the ratio is measured by scanning across the water lines. In this case, it is possible to adjust for the saturation effects by using a calibration measurement. In the case of the on-line measurements, the ratio trend presented here does not match what is expected from calculations, although the values are reproducible. It is possible to calibrate the on-line ratio as a function of temperature, and use this calibration for further temperature measurements. However, when using a calibration measurement it is essential to keep all important parameters constant between the calibration measurement and the actual measurement. As long as the reason for the deviation from the predicted trend is not understood, it is not possible to control the cause of this effect when going from the calibration measurement to the actual measurement. The only difference between the results measured by scanning and with the on-line approach is the operation of the laser, but we have not been able to pinpoint which factor is the cause of the different results. It is possible that this is a result of the inherent instability of the multi-mode laser, and that it could be avoided using a single-mode laser system with better wavelength tuning precision.

Despite the need to understand and calibrate the saturation behavior, the WALTHER method has several advantages. The intensity ratio of the H_2_O line groups II and III is very sensitive to small temperature changes in the interval 1000–2000 K, making this a very sensitive technique for small temperature changes. Utilizing the absorption of water, which is almost always present in relatively high concentrations in combustion, means there is no need for seeding of other species into the flame. The mid-infrared laser beams are much less sensitive to scattering compared to visible and ultraviolet wavelengths, making this a viable technique for temperature measurements in sooty flames and other high-scattering environments. In addition, mid-IR DFWM is a promising candidate for quantitative concentration measurements of molecules in combustion environments,^4^ and in those types of measurements this technique can be applied with the same setup without needing any extra complex instrumentation, apart from some type of calibration measurement.

In conclusion, measuring the water line ratio using scanning over a less than 1 cm^–1^ spectral range can provide accurate temperature measurements when combined with the appropriate calibration measurements. The on-line approach needs to be further investigated as it does not seem to provide completely reliable results. However, if the practical challenges of the on-line approach can be identified and addressed, it would become a useful tool for studying time-varying combustion phenomena.

## References

[1] Kohse-Höinghaus K. Barlow R.S. Aldén M. Wolfrum W. “Combustion at the Focus: Laser Diagnostics and Control”. Proc. Combust. Inst. 2005 30(1): 89–123.

[2] Sun Z.W. Li Z.S. Li B. Aldén M. “Flame Temperature Diagnostics with Water Lines Using Mid-Infrared Degenerate Four-Wave Mixing”. J. Raman Spectrosc. 2011 42(10): 1828–1835.

[3] Sun Z.W. Li Z.S. Li B. Alwahabi Z. Aldén M. “Quantitative C_2_H_2_ Measurements in Sooty Flames Using Mid-Infrared Polarization Spectroscopy”. Appl. Phys. B: Lasers Opt. 2010 101(1): 423–432.

[4] Hot D. Pedersen R.L. Weng W. Zhang Y. Aldén M. Li Z.S. “Spatially and Temporally Resolved IR-DFWM Measurement of HCN Released from Gasification of Biomass Pellets”. Proc. Combust. Inst. 2019 37(2): 1337–1344.

[5] Rothman L.S. Gordon I.E. Barber R.J. Dothe H. Gamache R.R. Goldman A. Perevalov V.I. Tashkun S.A. Tennyson J. “HITEMP, the High-Temperature Molecular Spectroscopic Database”. J. Quant. Spectrosc. Radiat. Transf. 2010 111(15): 2139–2150.

[6] Kochanov R.V. Gordon I.E. Rothman L.S. Wcisło P. Hill C. Wilzewski J.S. “HITRAN Application Programming Interface (hapi): A Comprehensive Approach to Working with Spectroscopic Data”. J. Quant. Spectrosc. Radiat. Transf. 2016 177: 15–30.

[7] Williams S. Zare R.N. Rahn L.A. “Reduction of Degenerate Four-Wave Mixing Spectra to Relative Populations II. Strong-Field Limit”. J.Chem. Phys. 1994 101(2): 1093–1107.

[8] Reichardt T.A. Lucht R.P. “Theoretical Calculation of Line Shapes and Saturation Effects in Polarization Spectroscopy”. J.Chem. Phys. 1998 109(14): 5830–5843.

[9] Reichardt T.A. Giancola W.C. Lucht R.P. “Experimental Investigation of Saturated Polarization Spectroscopy for Quantitative Concentration Measurements”. Appl. Opt. 2000 39(12): 2002–2008. 18345100 10.1364/ao.39.002002

[10] Bratfalean R.T. Lloyd G.M. Ewart P. “Degenerate Four-Wave Mixing for Arbitrary Pump and Probe Intensities”. J. Opt. Soc. Am. B. 1999 16(6): 952–960.

[11] Lucht R.P. Farrow R.L. Rakestraw D.J. “Saturation Effects In Gas-phase Degenerate Four-wave-mixing Spectroscopy – Nonperturbative Calculations”. J. Opt. Soc. Am. B. 1993 10(9): 1508–1520.

[12] Bultitude K. Bratfalean R. Ewart P. “Saturation Effects in Molecular Spectroscopy Using Degenerate Four-Wave Mixing”. J. Raman Spectrosc. 2003 34(12): 1030–1036.

[13] Reichardt T.A. Lucht R.P. “Interaction of Closely Spaced Resonances in Degenerate Four-Wave-Mixing Spectroscopy”. J. Opt. Soc. Am. B. 1997 14(10): 2449–2458.

[14] Zhao F.Q. Hiroyasu H. “The Applications of Laser Rayleigh Scattering to Combustion Diagnostics”. Prog. Energ. Combust. Sci. 1993 19(6): 447–485.

[15] Sutton J.A. Driscoll J.F. “Rayleigh Scattering Cross Sections of Combustion Species at 266, 355, and 532 nm for Thermometry Applications”. Opt. Lett. 2004 29(22): 2620–2622. 15552664 10.1364/ol.29.002620

[16] Fielding J. Frank J.H. Kaiser S.A. Smooke M.D. Long M.B. “Polarized/Depolarized Rayleigh Scattering for Determining Fuel Concentrations in Flames”. Proc. Combust. Inst. 2002 29(2): 2703–2709.

[17] Zetterberg J. Li Z.S. Afzelius M. Aldén M. “Two-Dimensional Temperature Measurements in Flames Using Filtered Rayleigh Scattering at 254 nm”. Appl. Spectrosc. 2008 62(7): 778–783. 18935828 10.1366/000370208784909526

[18] Peck E.R. Khanna B.N. “Dispersion of Nitrogen*”. J. Opt. Soc. Am. 1966 56(8): 1059–1063.

[19] Zhang J. Lu Z.H. Wang L.J. “Precision Refractive Index Measurements of Air, N_2_, O_2_, Ar, and CO_2_ with a Frequency Comb”. Appl. Opt. 2008 47(17): 3143–3151. 18545287 10.1364/ao.47.003143

[20] Gardiner W. Hidaka Y. Tanzawa T. “Refractivity of Combustion Gases”. Combust. Flame. 1981 40: 213–219.

[21] Old J.G. Gentili K.L. Peck E.R. “Dispersion of Carbon Dioxide*”. J. Opt. Soc. Am. 1971 61(1): 89–90.

[22] Li Z.S. Rupinski M. Zetterberg J. Alwahabi Z.T. Aldén M. “Mid-Infrared Polarization Spectroscopy of Polyatomic Molecules: Detection of Nascent CO_2_ and H_2_O in Atmospheric Pressure Flames”. Chem. Phys. Lett. 2005 407(4): 243–248.

[23] Pedersen R.L. Li Z.S. “Infrared Degenerate Four-Wave Mixing with Upconversion Detection for Quantitative Gas Sensing”. J. Vis. Exp. 2019 145: e59040. 10.3791/59040 30958481

[24] Dam J.S. Tidemand-Lichtenberg P. Pedersen C. “Room-temperature mid-infrared single-photon spectral imaging”. Nat. Photonics. 2012 6(11): 788–793.

[25] Høgstedt L. Dam J.S. Sahlberg A.L. Li Z.S. Aldén M. Pedersen C. Tidemand-Lichtenberg P. “Low-Noise Mid-IR Upconversion Detector for Improved IR-Degenerate Four-Wave Mixing Gas Sensing”. Opt. Lett. 2014 39(18): 5321–5324. 26466261 10.1364/OL.39.005321

[26] S. Gordon, B.J. McBride. “Nasa Reference Publication 1311: Computer Program for Calculation of Complex Chemical Equilibrium Compositions and Applications”. National Aeronautics and Space Administration. (1996). https://www.grc.nasa.gov/WWW/CEAWeb/RP-1311P2.htm [accessed Sep 8 (2020)].

[27] Kristensson E. Ehn A. Bood J. Aldén M. “Advancements in Rayleigh Scattering Thermometry by Means of Structured Illumination”. Proc. Combust. Inst. 2015 35(3): 3689–3696.

[28] Kempema N.J. Long M.B. “Quantitative Rayleigh Thermometry for High Background Scattering Applications with Structured Laser Illumination Planar Imaging”. Appl. Opt. 2014 53(29): 6688–6697. 25322370 10.1364/AO.53.006688

[29] Wang G.H. Clemens N.T. Varghese P.L. “Two-Point, High-Repetition-Rate Rayleigh Thermometry in Flames: Techniques to Correct for Apparent Dissipation Induced by Noise”. Appl. Opt. 2005 44(31): 6741–6751. 16270563 10.1364/ao.44.006741

[30] Miles R.B. Lempert W.R. Forkey J.N. “Laser Rayleigh Scattering”. Meas. Sci. Technol. 2001 12(5): R33–R51.

